# Low Lymphocyte-to-Monocyte Ratio as a Possible Predictor of an Unfavourable Clinical Outcome in Patients with Acute Ischemic Stroke after Mechanical Thrombectomy

**DOI:** 10.1155/2022/9243080

**Published:** 2022-12-10

**Authors:** Katarína Pinčáková, Georgi Krastev, Jozef Haring, Miroslav Mako, Viktória Mikulášková, Vladimír Bošák

**Affiliations:** ^1^Department of Laboratory Medicine, Faculty of Health and Social Care, Trnava University in Trnava, 918 43 Trnava, Slovakia; ^2^Department of Haematology, Faculty Hospital Trnava, 917 75 Trnava, Slovakia; ^3^Jessenius Medical Faculty in Martin, Comenius University in Bratislava, 036 01 Martin-Záturčie, Slovakia; ^4^Department of Neurology, Faculty Hospital Trnava, 917 75 Trnava, Slovakia; ^5^Faculty of Medicine, Comenius University in Bratislava, 813 72 Bratislava, Slovakia

## Abstract

**Background:**

Although considerable progress has been made in the treatment of acute ischemic stroke (AIS), the clinical outcome of patients is still significantly influenced by the inflammatory response that follows stroke-induced brain injury. The aim of this study was to evaluate the potential use of complete blood count parameters, including indices and ratios, for predicting the clinical outcome in AIS patients undergoing mechanical thrombectomy (MT).

**Methods:**

This single-centre retrospective study is consisted of 179 patients. Patient data including demographic characteristics, risk factors, clinical data, laboratory parameters on admission, and clinical outcome were collected. Based on the clinical outcome assessed at 3 months after MT by the modified Rankin Scale (mRS), patients were divided into two groups: the favourable group (mRS 0–2) and unfavourable group (mRS 3–6). Stepwise multivariate logistic regression analysis was used to detect an independent predictor of the unfavourable clinical outcome.

**Results:**

An unfavourable clinical outcome was detected after 3 months in 101 patients (54.4%). Multivariate logistic regression analysis confirmed that the lymphocyte-to-monocyte ratio (LMR) was an independent predictor of unfavourable clinical outcome at 3 months (odds ratio = 0.761, 95% confidence interval 0.625–0.928, and *P* = 0.007). The value of 3.27 was chosen to be the optimal cut-off value of LMR. This value could predict the unfavourable clinical outcome with a 74.0% sensitivity and a 54.4% specificity.

**Conclusion:**

The LMR at the time of hospital admission is a predictor of an unfavourable clinical outcome at 3 months in AIS patients after MT.

## 1. Introduction

Acute ischemic stroke (AIS) accounts for more than 60% of all incident strokes [1]. In cases of AIS with large artery occlusions, the standard treatment method is mechanical thrombectomy (MT) with or without previous intravenous thrombolysis using the recombinant tissue plasminogen activator. Although the treatment window for MT has recently been extended through the use of modern imaging techniques, and the level of successful recanalization has reached more than 80%, the clinical outcome and final prognosis of patients are still dependent on other factors [2, 3]. Based on the current knowledge about the complex pathophysiology of AIS, one crucial prognostic factor for the clinical outcome of AIS patients is the systemic inflammatory response of the organism [4]. Disruption of the integrity of the blood-brain barrier (BBB) caused by stroke-related brain injury and subsequent release of chemoattractants into the bloodstream lead to recruitment of stimulated peripheral leukocytes into the ischemic tissue [5]. The innate immune system responds by initially sending neutrophils to the damaged brain tissue. However, the neutrophils contribute to secondary damage in the brain parenchyma through the production of inducible nitric oxide synthase, metalloproteinases (MPs), and reactive oxygen species (ROS) [6, 7]. A few hours after the release of the neutrophils, monocytes invade the ischemic tissue and exacerbate the tissue damage further through the secretion of proinflammatory cytokines, such as tumour necrosis factor-*α* (TNF-*α*), interleukin (IL)-1*β*, and IL-6 [8]. In contrast, severe diseases like stroke or sepsis can activate the hypothalamus-pituitary axis, thereby elevating cortisol levels, which lead to the induction of apoptosis of lymphocytes and subsequent attenuation of proinflammatory responses and regulation of immunological reactions [9, 10]. Ultimately, the inflammatory response following AIS is characterized by increased numbers of peripheral neutrophils and monocytes and decreased numbers of lymphocytes, which can be easily monitored by complete blood count analysis. Although each of the individual parameters can be influenced by confounding factors, ratios of these parameters are currently believed to be more stable and have better predictive value. The most well-established ratios, which serve as markers of inflammation, include the lymphocyte-to-monocyte ratio (LMR) (or reversely monocyte-to-lymphocyte ratio) and the neutrophil-to-lymphocyte ratio (NLR). The diagnostic and prognostic utilities of these ratios have been observed in several inflammation-related diseases, including cancer, type 2 diabetes mellitus (T2DM), diabetic kidney injury, nonalcoholic fatty liver disease, inflammatory bowel disease, epilepsy, Guillain-Barré syndrome, and COVID-2019 [9, 11–17]. Furthermore, according to recent studies, these parameters are predictive markers for the development of poststroke complications, including early neurological deterioration and poststroke depression [5, 18]. Moreover, from a practical point of view, a complete blood count analysis is one of the quickest, simplest, most accessible, and most economical laboratory tests. Therefore, the aim of this study was to investigate the prognostic value of haemogram parameters, their indices, and ratios including LMR and NLR, in patients with AIS undergoing MT.

## 2. Materials and Methods

### 2.1. Study Design and Patient Selection

A single-centre retrospective cohort study of all consecutive AIS patients who underwent MT between June 2016 and July 2021 was conducted at the Cerebrovascular Stroke Centre of Faculty Hospital Trnava, Trnava, Slovakia. Patients who met the following inclusion criteria were enrolled in the study: (1) age ≥ 18 years old; (2) AIS with neurologic impairment caused by large vessel occlusion within 24 hours of onset; (3) presence of large vessel occlusion in anterior or posterior circulation, verified by either computed tomography angiography, magnetic resonance angiography, or digital subtraction angiography; (4) received MT.

**Figure 1:**
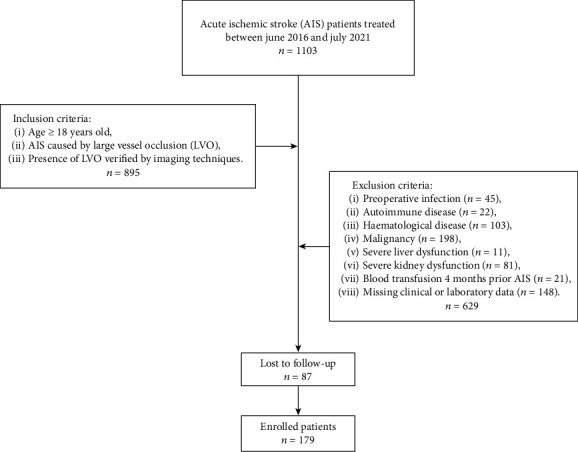
Flow chart of patient selection.

In all patients, MT was conducted in compliance with the guidelines of the American Heart Association/American Stroke Association [19]. The exclusion criteria were as follows: (1) patients with a preoperative infection, an autoimmune or a haematological disease, history of malignancy, severe liver, or kidney dysfunction; (2) patients who had received a blood transfusion 4 months prior to the AIS; (3) patients with missing clinical or laboratory data; (4) patients who were lost to follow-up. A preoperative infection was defined by the evidence of active infection such as fever, significantly increased white blood cells (>20 × 10^9^/L), or typical clinical manifestations. A flow chart of patient selection is shown in Figure 1.

This study was approved by the Ethical Committee of the Faculty Hospital Trnava conforming to the Declaration of Helsinki. Informed patient consent was not required due to the retrospective nature of this study.

### 2.2. Data Collection and Definitions

Patient data including demographic characteristics, risk factors, clinical data, and laboratory parameters were collected on admission. The demographic characteristics were comprised of age and gender. The risk factors included hypertension, coronary heart disease, atrial fibrillation, T2DM, previous stroke or transient ischemic attack (TIA), and dyslipidaemia. The clinical data consisted of stroke severity on admission, stroke aetiology, occlusion site, intravenous thrombolysis (IVT), and vascular recanalization. The stroke severity on admission was determined using the National Institute of Health Stroke Scale (NIHSS) score [20]. The stroke aetiology was established based on the Trial of 10172 in Acute Stroke Treatment criteria [21]. The sites of occlusion were categorized as internal carotid artery, carotid T, middle cerebral artery, posterior cerebral artery, vertebral artery, or basilar artery. In selected patients, IVT was administered before MT, following the established guidelines. Vascular recanalization was evaluated using the Thrombolysis in Cerebral Infarction (TICI) scale at the end of MT, and a successful recanalization was defined as a TICI 2b or 3.

### 2.3. Laboratory Measurements

Blood samples were drawn from the antecubital vein in the emergency room immediately after hospital admission. A complete blood count analysis was performed on the peripheral venous blood samples in a tube containing dipotassium ethylenediaminetetraacetic acid within 30 minutes of admission using a Siemens Advia 2120 automated haematology analyser (Siemens Healthineers, Erlangen, Germany). A complete blood count was consisted of white blood cell count, neutrophil count, lymphocyte count, monocyte count, red blood cell count, haemoglobin, haematocrit, mean corpuscular volume, red blood cell distribution width coefficient of variation (RDW-CV), platelet count, mean platelet volume, platelet distribution width, plateletcrit, NLR, LMR, platelet-to-white blood cell ratio, platelet-to-neutrophil ratio, platelet-to-lymphocyte ratio, mean platelet volume-to-platelet ratio, platelet distribution width-to-platelet ratio, and red blood cell distribution width-to-platelet ratio. NLR was calculated by dividing the neutrophil count by the lymphocyte count. LMR was calculated by dividing the lymphocyte count by the monocyte count. Platelet-to-white blood cell ratio was calculated by dividing the platelet count by the white blood cell count. Platelet-to-neutrophil ratio was calculated by dividing the platelet count by neutrophil count. Platelet-to-lymphocyte ratio was calculated by dividing the platelet count by lymphocyte count. Mean platelet volume-to-platelet ratio was calculated by dividing the mean platelet volume by the platelet count. Platelet distribution width-to-platelet ratio was calculated by dividing the platelet distribution width by the platelet count. Red blood cell distribution width-to-platelet ratio was calculated by dividing the RDW-CV by the platelet count.

### 2.4. Clinical Outcome

The clinical outcome assessed at 3 months after AIS by the modified Rankin Scale (mRS) was selected as the monitored outcome. A favourable clinical outcome was defined as an mRS score of 0–2. An unfavourable clinical outcome was defined as an mRS score of 3–6. The follow-up of mRS at 3 months was carried out by either conducting a simplified structured mRS questionnaire through a phone conversation with patients or their family members, or by outpatient visits [22].

### 2.5. Statistical Analysis

All patients were dichotomized according to the mRS score at 3 months (favourable 0–2 vs. unfavourable 3–6). The normal distribution was evaluated according to the Anderson-Darling test. The continuous variables that followed the normal distribution were expressed as mean and standard deviation. The continuous variables that were not subjected to normal distributions were presented as median and interquartile range. The categorical variables were expressed as frequency (*n*) and percentage (%). The difference between the two groups was analysed using the Student's *t*-test for normally distributed continuous variables or the Mann–Whitney *U* test for nonparametrically distributed continuous variables. The difference between the two groups of categorical variables was determined using the Chi-square test of the Fisher exact test. Associations between the clinical outcome and the variables were examined by univariate logistic regression analysis. Variables with *P* < 0.05 in univariate logistic regression analysis were then entered into a stepwise multivariate logistic regression analysis, to select the independent predictors of the unfavourable clinical outcome after MT. The receiver-operating characteristic curve analysis was performed, and the maximum Youden index was determined to define the optimal cut-off value for discrimination of clinical outcome after MT. The two-tailed value of *P* < 0.05 was considered to indicate a significant difference. All statistical analyses were performed using the Minitab 20.2.0 Statistical Software (Minitab, LLC, State College, PA, USA).

## 3. Results

### 3.1. Baseline Characteristics

A total of 895 consecutive patients that had been diagnosed with AIS and underwent MT were screened. Of these patients, 716 patients that met the exclusion criteria were ruled out. Finally, a total of 179 patients were enrolled in this study. The median age of all patients was 72.00 (69.00–81.00), and 89 (49.7%) were female. According to the mRS score at 3 months, patients were divided into a favourable (*n* = 78, 43.6%) or unfavourable (*n* = 101, 56.4%) group. Baseline data for both groups are summarized in Table 1.

Patients in the unfavourable group were significantly older (*P* < 0.001) and were more likely to be female than male (*P* = 0.040). The unfavourable group had a significantly higher occurrence of hypertension (*P* = 0.002), coronary heart disease (*P* = 0.002), and atrial fibrillation (*P* = 0.005). In the group with an unfavourable clinical outcome, patients were more likely to have a significantly higher NIHSS score on admission (*P* < 0.001), higher rate of carotid T-type occlusion (*P* < 0.001), lower rate of middle cerebral artery M2 segment occlusion (*P* = 0.010), and an increased incidence of cardioembolic stroke (*P* = 0.033). In terms of laboratory findings, patients in the unfavourable group were found to have a significantly lower lymphocyte count (*P* = 0.009), LMR value (*P* = 0.008), and platelet-to-neutrophil ratio value (*P* = 0.046). At the same time, RDW-CV values were significantly decreased in the favourable group (*P* = 0.023). Other clinical data and laboratory parameters between the two groups showed no significant differences (Table 2).

### 3.2. Univariate and Multivariate Logistic Regression Analyses to Assess Clinical Outcome

Univariate logistic regression analysis revealed that the following independent variables were significantly associated with an unfavourable clinical outcome at 3 months: age (*P* < 0.001), gender (*P* = 0.042), hypertension (*P* = 0.002), coronary artery disease (*P* = 0.002), atrial fibrillation (*P* = 0.006), T2DM (*P* = 0.012), previous history of stroke/TIA (*P* = 0.010), higher NIHSS score on admission (*P* < 0.001), cardioembolic stroke (*P* = 0.034), and carotid T-type occlusion (*P* = 0.002). With respect to the laboratory parameters, lower lymphocyte count and lower LMR values were significantly associated with an unfavourable clinical outcome (*P* = 0.020 and *P* = 0.037, respectively) (Table 3).

Stepwise multivariate logistic regression analysis showed that LMR values were an independent predictor of the unfavourable clinical outcome at 3 months (odds ratio = 0.761, 95% confidence interval 0.625–0.928, and *P* = 0.007). In addition, higher age, T2DM, previous history of stroke/TIA, higher NIHSS score on admission, and carotid T-type occlusion also presented as independent risk factors for predicting an unfavourable outcome (Table 4).

**Figure 2:**
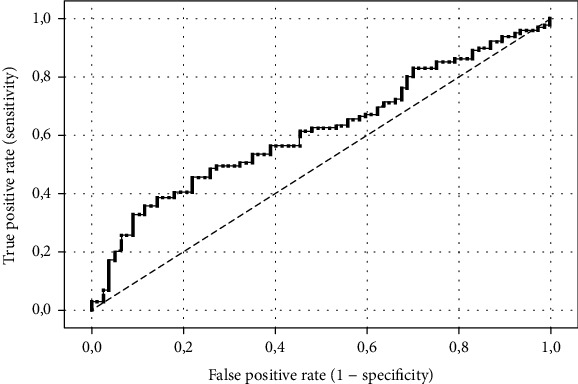
Receiver-operating characteristic curve of LMR predicting values of the 3-month unfavourable outcome in patients with AIS undergoing MT.

According to the receiver-operating characteristic curve analysis, the optimal cut-off value of LMR levels that predicted the 3-month unfavourable outcome of patients with AIS who underwent MT was 3.27. The area under the curve of LMR was calculated as 0.616 (95% confidence interval 0.745–0.993), with a sensitivity of 74.0%, specificity of 54.5%, and Youden index of 0.28 (Figure 2).

## 4. Discussion

Arterial occlusion results in immediate reduction of blood flow in the brain, which leads to excitotoxicity and ionic imbalance, followed by neuronal cell death in hypoperfused regions [23]. These events are followed by the release of heat shock proteins, nucleotides, adenosine triphosphate, hypoxia-inducible factor-1*α*, high mobility group box 1 (HMGB-1), S100 proteins, and heparan sulphate, which are known as danger-associated molecular patterns (DAMPs). DAMPs bind to pattern recognition receptors to activate brain-resident microglial and endothelial cells in the brain parenchyma, which lead to the release of proinflammatory cytokines such as IL-1*β*, IL-6, IL-17, IL-18, and TNF-*α*, as well as ROS and matrix MPs [24, 25]. This leads to the expression of E-selectin, P-selectin, intercellular adhesion molecule-1, and vascular cell adhesion molecule-1 on the surface of endothelial cells [26], which results in disruption of the integrity of BBB, release of chemoattractants into the circulation, and recruitment of peripheral immune cells into the ischemic tissue [27].

Infiltration of the peripheral immune cells occurs in a precise order. Neutrophils are the first to infiltrate the site in response to the increased expression of chemoattractants, such as chemokine-like factor 1, C-X-C motif chemokine ligand 1, and C-X-C motif chemokine ligand 2. The concentration of neutrophils in the brain parenchyma increases 30 minutes after ischemia has occurred and reaches its peak between the first and third day, after which levels slowly decrease until the seventh day [7, 8, 28]. The neutrophils produce inducible nitric oxide synthase, MPs, and ROS, which potentiate the disruption of the integrity of the BBB and contribute to secondary damage in the brain parenchyma [7]. The production of neutrophil extracellular traps further activates local thrombocytes, which links individual parts of the pathophysiological process of AIS [29]. Approximately 4 to 6 hours after neutrophil infiltration, monocytes invade the ischemic tissue in response to monocyte chemoattractant protein-1. The increase in monocytes occurs during the first day, with the highest concentration observed between 3 to 7 days after AIS [30]. The exacerbation of tissue damage in the hyperacute and acute phase of AIS is mediated predominantly by CD14^high^CD16^−^CCR2^high^ monocytes that secrete TNF-*α*, IL-1*β*, and IL-6. Monocyte levels in the brain circulation after AIS return to normal levels after approximately 2 weeks [7, 8, 27].

Increased accumulation of neutrophils and monocytes in the brain parenchyma is similar to that observed in the circulation. Increased levels of circulating monocytes are caused by fast contraction of lymphatic organs as part of the acute response to AIS [28]. Increased levels of peripheral neutrophils correspond to an increased release from the bone marrow and reduction in apoptosis [31]. In contrast to neutrophils and monocytes, lymphocytes infiltrate the ischemic tissue at significantly lower concentrations. Destruction of the BBB is mediated by Th17, *γδ*T cells, and CD8^+^ T cells, primarily through the secretion of proinflammatory cytokines IL-2, IL-17, IL-21, IL-22, interferon gamma, and TNF-*α* [32]. The migration of T-lymphocytes into the brain parenchyma begins during the first 24 hours, peaking on the third day [16]. Of all the leukocytes, T-lymphocytes persist in the brain tissue the longest, and similar to monocytes, are involved in tissue repair during later phases of the stroke [33]. The number of peripheral lymphocytes decreases exponentially for up to seven days following AIS, with the lowest level occurring after 12 hours [8]. This dramatic decrease is caused by activation of the hypothalamus-pituitary axis and sympathetic nervous system [34]. Although the specific molecular mechanism has yet to be elucidated, studies have indicated that one of the DAMPs, HMGB-1, plays an important role during the process of immunosuppression following AIS. HMGB-1 induces the release of immature monocytes from the bone marrow after binding to the receptor of advanced glycation end-product. Once released into the circulation, the immature monocytes are characterized by lower expression of major histocompatibility complex class II molecules, decreased secretion of cytokines TNF-*α* and IL-10, and reduced levels of antigen presentation. Inadequate costimulatory signals to the lymphocytes promote T-cell dysfunction and apoptosis. Furthermore, inhibition and activation via arginase 1 can also lead to the induction of apoptosis in lymphocytes [35–37]. These actions are followed by an increase in catecholamine and cortisol levels as a result of acute physiological stress and lead to the inhibition of antigen presentation by *β*2-adrenoceptors and a decrease in inflammatory cytokines by antigen-presenting cells [24].

Immunopathogenesis is one of the reasons why AIS patient prognosis studies focus on the number of peripheral leukocytes. Even though multiple studies have previously confirmed the importance of increased numbers of neutrophils and monocytes and decreased numbers of lymphocytes at the time of admission in the prognosis of short-term unfavourable clinical outcomes [38–40], currently, the ratios are believed to offer more predictive values and are therefore favoured over the individual parameters. In the current retrospective observational study, we confirmed that the LMR can act as an independent risk factor of the unfavourable clinical outcome in AIS patients that undergo MT. Although Ren et al. were the first to describe the relationship between lower LMR values at admission and poor prognosis in AIS patients, their initial study did not take treatment strategies into consideration [41]. Shortly after, a follow-up study by Ren et al. confirmed the prognostic importance of LMR levels in AIS patients that had been treated with IVT. In their study, the optimal cut-off level of LMR was 3.48, which is very close to the defined cut-off level in our study (3.27) [42]. Lux et al. were the first to focus on the relationship between LMR and prognosis in patients who underwent MT. In addition to LMR levels at admission, this study also examined the dynamic development of this parameter over time and reported a significant association between LMR levels at 24 hours after MT and poorer clinical outcome [43]. Park et al. focused on a more significant time gap and examined LMR levels in patients with significantly worse clinical outcomes [36]. Their findings confirmed the increasing significance of the immunosuppressive state following stroke-induced brain injury for the prediction of the short-term clinical outcome. More recently, Oh et al. studied patients treated with MT with previous IVL and found that LMR levels that were lower than the defined cut-off (2.5) were not only independent predictive factors of the unfavourable 3-month mRS score but also predicted symptomatic intracerebral haemorrhage [44]. These findings supported the work by Song et al., who reported that levels of LMR in the lowest tertile (≤3.12) were connected to a significant risk of haemorrhagic transformation [45].

To the best of our knowledge, our study is the first to describe a connection between a 3-month functional outcome in patients with AIS who underwent MT and haemogram parameters, including indices and ratios. The main limitation of our study was its design as a single-centre retrospective study, which led to the exclusion of a large number of patients and possible selection bias. In addition, none of the information about poststroke complications was taken into account, which could potentially contribute to the unfavourable clinical outcome in patients. We also did not measure the dynamic change in LMR during hospitalization.

## 5. Conclusions

In the current study, we have confirmed the importance of LMR as a potential biomarker for assessing the risk of an unfavourable clinical outcome before MT in patients with AIS. Together with previous studies by other groups, our findings indicate that using LMR as a predictor of clinical outcome could simplify the complex decision-making process of interventional radiologists between the benefits and risks of MT in patients with AIS.

## Figures and Tables

**Table 1:** Baseline characteristics of patient study groups on the basis of 3-month outcome.

Characteristics	Patients (*n* = 179)	Favourable group (*n* = 78)	Unfavourable group (*n* = 101)	*P*
Demographics				
Age (years)	72.00 (69.00–81.00)	69.00 (64.25–75.00)	77.00 (69.00–83.00)	<0.001
Female, *n* (%)	89 (49.7)	32 (41.0)	57 (56.4)	0.040
Risk factors				
Hypertension, *n* (%)	141 (78.8)	53 (68.0)	88 (87.1)	0.002
Coronary heart disease, *n* (%)	71 (39.7)	21 (26.9)	50 (49.5)	0.002
Atrial fibrillation, *n* (%)	59 (33.0)	17 (21.8)	42 (41.6)	0.005
Type 2 diabetes mellitus, *n* (%)	47 (26.3)	13 (16.7)	34 (33.7)	0.009
Stroke/TIA, *n* (%)	26 (14.5)	5 (6.4)	21 (20.8)	0.005
Dyslipidaemia, *n* (%)	24 (13.4)	8 (10.3)	16 (15.8)	0.272
Clinical data				
Admission NIHSS	15.00 (9.00–21.00)	10.00 (6.75–16.25)	18.00 (13.00–22.00)	<0.001
Aetiology, *n* (%)				
Large artery atherosclerosis	78 (43.6)	38 (48.7)	40 (39.6)	0.223
Cardioembolic	85 (47.5)	30 (38.5)	46 (45.5)	0.033
Others	16 (8.9)	10 (12.8)	6 (5.9)	0.111
Occlusion site, *n* (%)				
ICA	3 (1.7)	1 (1.3)	2 (1.9)	0.715
Carotid T	26 (14.5)	3 (3.9)	23 (22.8)	<0.001
MCA M1	58 (32.4)	27 (34.6)	31 (30.7)	0.579
MCA M2	51 (28.5)	30 (38.5)	21 (20.8)	0.010
ICA + MCA	16 (8.9)	5 (6.4)	11 (10.9)	0.290
PCA	13 (7.3)	8 (10.3)	5 (5.0)	0.177
VA	1 (0.6)	1 (1.3)	0 (0.0)	0.737
BA	11 (6.2)	3 (3.9)	8 (7.9)	0.249
Left hemisphere, *n* (%)	99 (55.3)	48 (61.5)	51 (50.5)	0.250
Thrombolysis, *n* (%)	76 (42.5)	38 (48.7)	38 (37.6)	0.137
Recanalization (TICI ≥ 2b), *n* (%)	168 (93.9)	75 (96.2)	93 (92.1)	0.249

Abbreviations: BA: basilar artery; ICA: internal carotid artery: MCA M1: middle cerebral artery M1 segment; MCA M2: middle cerebral artery M2 segment; NIHSS: National Institutes of Health Stroke Scale; PCA: posterior cerebral artery; TIA: transient ischemic attack; TICI: thrombolysis in cerebral infarction; VA: vertebral artery.

**Table 2:** Laboratory parameters of patient study groups on the basis of 3-month outcome.

Laboratory parameters	Patients (*n* = 179)	Favourable group (*n* = 78)	Unfavourable group (*n* = 101)	*P*
WBC (×10^9^/L)	8.25 (6.84–10.31)	8.32 (6.99–9.89)	8.24 (6.82–10.90)	0.599
Neutrophil count (×10^9^/L)	6.28 ± 2.74	5.88 ± 2.08	6.59 ± 3.13	0.076
Lymphocyte count (×10^9^/L)	1.60 (1.18–2.31)	1.81 (1.31–2.44)	1.47 (1.03–2.07)	0.009
Monocyte count (×10^9^/L)	0.44 (0.35–0.58)	0.43 (0.35–0.51)	0.45 (0.33–0.60)	0.134
RBC (×10^12^/L)	4.47 ± 0.44	4.53 ± 0.41	4.43 ± 0.46	0.139
Haemoglobin (g/L)	140.35 ± 15.39	141.76 ± 14.52	139.27 ± 16.02	0.279
Haematocrit	0.42 ± 0.04	0.42 ± 0.04	0.41 ± 0.04	0.430
MCV (Fl)	93.13 ± 4.66	92.77 ± 4.63	93.41 ± 4.69	0.364
RDW-CV (%)	13.40 (13.00–14.25)	13.30 (12.80–13.97)	13.70 (13.10–14.40)	0.023
PLT (×10^9^/L)	222.17 ± 55.62	230.56 ± 57.97	215.56 ± 53.06	0.078
MPV (Fl)	9.50 (8.00–10.70)	9.35 (7.60–10.50)	9.60 (8.25–10.70)	0.067
PDW (%)	55.83 ± 6.61	54.62 ± 6.16	56.94 ± 6.87	0.091
Plateletcrit	0.20 (0.17–0.24)	0.21 (0.18–0.24)	0.20 (0.16–0.24)	0.339
NLR	3.26 (2.05–5.17)	3.02 (1.92–4.42)	3.78 (2.25–5.65)	0.077
LMR	3.83 (2.59–5.24)	4.20 (3.16–5.72)	3.50 (2.21–5.06)	0.008
PLT/WBC ratio	26.97 ± 8.92	28.42 ± 8.59	25.85 ± 9.05	0.055
PNR	38.92 (26.96–52.89)	41.15 (29.46–54.21)	35.90 (24.09–49.56)	0.046
PLR	126.57 (94.24–182.55)	126.99 (91.19–164.37)	123.73 (95.27–203.70)	0.629
MPV/PLT ratio	0.04 (0.03–0.06)	0.04 (0.03–0.05)	0.04 (0.04–0.06)	0.155
PDW/PLT ratio	0.26 ± 0.09	0.25 ± 0.08	0.28 ± 0.09	0.083
RDW/PLT ratio	0.06 (0.05–0.08)	0.06 (0.05-0.08)	0.06 (0.05–0.08)	0.091

Abbreviations: Fl: femtoliter; LMR: lymphocyte-to-monocyte ratio; MCV: mean corpuscular volume; MPV: mean platelet volume; MPV/PLT ratio: mean platelet volume-to-platelet ratio; NLR: neutrophil-to-lymphocyte ratio; PDW: platelet distribution width; PDW/PLT ratio: platelet distribution width-to-platelet ratio; PLR: platelet-to-lymphocyte ratio; PLT: platelet count; PLT/WBC ratio: platelet-to-white blood cell ratio; PNR: platelet-to-neutrophil ratio; RBC: red blood cell count; RDW-CV: red blood cell distribution width coefficient of variation; RDW/PLT ratio: red blood cell distribution width-to-platelet ratio; WBC: white blood cell count.

**Table 3:** Univariate logistic regression analysis for an unfavourable outcome.

	OR	95% CI	*P*
Age	1.104	1.062–1.148	<0.001
Female	1.862	1.024–3.388	0.042
Hypertension	3.193	1.506–6.772	0.002
Coronary heart disease	2.661	1.411–5.019	0.002
Atrial fibrillation	2.554	1.310–4.979	0.006
Diabetes mellitus	2.537	1.230–5.240	0.012
Stroke/TIA	3.833	1.374–10.688	0.010
Admission NIHSS	1,134	1.078–1.192	<0.001
Cardioembolic	1.913	1.049–3.490	0.034
Carotid T	7.372	2.124–25.580	0.002
Lymphocyte count (×10^9^/L)	0.965	0.935–0.995	0.020
RDW-CV (%)	1.342	0.980–1.836	0.060
LMR	0.860	0.745–0.993	0.037
PNR	0.834	0.645–1.079	0.165

Abbreviations: CI: confidence interval; LMR: lymphocyte-to-monocyte ratio; NIHSS: National Institutes of Health Stroke Scale; OR: odds ratio; PNR: platelet-to-neutrophil ratio; RDW-CV: red blood cell distribution width coefficient of variation; TIA: transient ischemic attack.

**Table 4:** Multivariate logistic regression model of predictors of an unfavourable outcome.

	OR	95% CI	*P*
Age	1.127	1.071–1.187	<0.001
Diabetes mellitus	3.182	1.240–8.163	0.016
Stroke/TIA	6.772	1.857–24.693	0.004
Admission NIHSS	1.155	1.079–1.126	<0.001
Carotid T	6.771	1.630–28.130	0.008
LMR	0.761	0.624–0.928	0.007

Abbreviations: CI: confidence interval; LMR: lymphocyte-to-monocyte ratio; NIHSS: National Institutes of Health Stroke Scale; OR: odds ratio; TIA: transient ischemic attack.

## Data Availability

The data used to support the findings of this study are available from the corresponding author upon request.
